# Sexual health literacy and education programs for culturally and linguistically diverse young people living in Australia, Canada, New Zealand, and the United Kingdom: a scoping review of content evaluations

**DOI:** 10.1093/her/cyaf056

**Published:** 2025-12-02

**Authors:** Ruth Wagstaff, Kirstie Daken, Amy B Mullens, Claire Moran, Zhihong Gu, Yibeltal Alemu, Judith A Dean

**Affiliations:** Institute for Resilient Regions, Centre for Health Research, School of Psychology and Wellbeing, University of Southern Queensland, 11 Salisbury Rd, Ipswich, Qld 4305, Australia; Faculty of Health - Psychology, Charles Darwin University, Ellengowan Dr, Casuarina, NT 0811, Australia; Institute for Resilient Regions, Centre for Health Research, School of Psychology and Wellbeing, University of Southern Queensland, 11 Salisbury Rd, Ipswich, Qld 4305, Australia; Institute for Resilient Regions, Centre for Health Research, School of Psychology and Wellbeing, University of Southern Queensland, 11 Salisbury Rd, Ipswich, Qld 4305, Australia; True Relationships and Reproductive Health (True), 230 Lutwyche Rd, Windsor, Qld 4030, Australia; Faculty of CI, Education & Social Justice, School of Education, Queensland University of Technology, 44 Musk Ave, Kelvin Grove, Qld 4059, Australia; Ethnic Communities Council of Queensland (ECCQ), 253 Boundary St, West End, Qld 4101, Australia; Faculty of Medicine, School of Public Health, The University of Queensland, 11 Wyndham St, Herston, Qld 4006, Australia; Faculty of Medicine, School of Public Health, The University of Queensland, 11 Wyndham St, Herston, Qld 4006, Australia

## Abstract

Young people from key priority communities, represent an important focus for enhancing sexual and reproductive health (SRH). There is an abundance of literature indicating that sexual health programs designed to improve sexual health literacy (SHL) for young people from culturally and linguistically diverse (CALD) backgrounds are often developed using established health behaviour models and community involvement models. However, little evidence exists on the processes or standards used to evaluate program effectiveness or update content to remain relevant and appealing. This scoping review of academic literature, and government and not-for-profit organizations reports in Australia, Canada, New Zealand, and the United Kingdom (countries with similar healthcare systems, immigration patterns and socioeconomic status), was conducted to determine if and how program content was reviewed and evaluated for currency and effectiveness. No academic or grey literature reports were identified that described the assessment of SHL program content or resources targeting young people from CALD backgrounds. Organizations and authors were contacted for further details of program content assessment with little success. This suggests a gap in the literature and lack of program evaluation or processes for updating of program content. This review highlights recommendations regarding advancing future research and enhancing program content evaluation.

## Introduction

In 2020, 25% of the 3.2 million young people aged 15–24 years living in Australia were born overseas [1]. In Australia, people who are born overseas in non-English speaking countries as well as those who do not speak English at home are often collectively referred to as people from culturally and linguistically diverse (CALD) backgrounds [2] and have been identified as a key priority population regarding sexual and reproductive health (SRH) [3]. In this paper, CALD will refer to people born overseas or who have one or more overseas-born parent, particularly from minority ethnic, migrant, or refugee backgrounds (hereafter referred to as CALD young people living in Australia, Canada, New Zealand and the United Kingdom). Attitudes and beliefs about sex can be barriers to help seeking for SRH issues among CALD populations, as sex-related topics may be considered as culturally sensitive or taboo [4]. Young people arriving in Australia from non-English speaking countries typically have limited knowledge of sexual health issues [4–6]. Their need for sexual health information is very important [7] as this is a key factor in helping them to achieve sexual health literacy (SHL); the knowledge, attitudes, and personal skills necessary to access, assess and apply information in making informed decisions regarding their sexual health and well-being [8, 9]. Informed consent, a key aspect of this broader concept, is critical for ensuring individuals have the autonomy and understanding to make decisions that align with their own health needs and values. However, they continue to experience a range of barriers such as language and other cultural factors (e.g. norms, values, and beliefs) to finding or seeking information that may contribute to sexual health inequities not experienced by other young people living in Australia [10].

UNESCO [11] recommends all sexual health services employ evidence-based collaboration frameworks to overcome barriers and identify enablers within local communities. Evidence-based frameworks serve to guide development and delivery of specific aspects of the program content. For example, the inclusion of program topics, content, and presentation approaches can be guided by evidence-based psychological theory (e.g. theory of reasoned action model) [12]. Evidence-based strategies to enhance skills such as communication, assertiveness, and problem solving need to be taught to and practiced by program participants particularly when content includes sensitive topics like intimate partner violence, and sexual health related matters such as condom use [13].

## Collaborative frameworks

Previous literature has described the use of evidence-based collaboration frameworks for the development of sexual health services and education/health promotion programs using action research [14–16] and the Delphi Method [17] across a wide range of communities and settings. However, it is important to note that while these methods provide valuable insights, they may not represent the highest quality evidence available. The value of evidence-based frameworks to design appropriate and effective sexual health education have been documented with First Nations peoples in Canada [18], New Zealand Aotearoa (NZ) [19], and Australia [20]. Other broader theoretical frameworks such as Bronfenbrenner’s Ecological Model [21] have also been used to effectively develop a deeper understanding of a given local culture, including barriers, enablers, and social systems [17] to enhance program design and quality. It is recommended that several evidence based guiding or theoretical frameworks are used in program design, content, teaching strategies, and resource development to enhance quality, however the key to quality programs is to ensure that evaluation frameworks are embedded as an integral part of these evidence-informed collaboration frameworks [22].

## Evaluation frameworks

Evaluation is the process of assessing the value and effectiveness of an intervention or program [23]. Evaluation conducted during program development is generally iterative, providing a mechanism for stakeholders to own the process of program development and delivery. It also ensures that the final program reflects stakeholder values, thereby maximizing desirable outcomes. In comparison, evaluation after the completion of program measures how effectively a program met the planned outcomes [24] and unplanned consequences [25]. Regardless of when evaluation occurs in the program planning and delivery cycle, the data and conclusions of evaluations may be used to justify ongoing program delivery, increasing outreach [26], and ongoing refinement of repeated programs [27].

In addition to stakeholder-desired outcomes, policies of authoritative bodies also shape evaluations by determining program outcomes. For example, UNESCO [11] report that outcomes of sexual health program evaluations include changes in sexual health knowledge, uptake of testing and diagnosis of sexually transmissible infections, HIV and other blood borne viruses (STI/HIV/BBV), and reduced rates of unplanned pregnancies [28], along with the intent to engage in safer sexual practices [29]. Such outcomes can be readily measured and understood and provide an indirect measure of relevance and acceptability of the programs. However, these outcomes do not necessarily inform acceptability of content to the target population, in this papers case young people from CALD backgrounds, their parents, or their broader communities; nor do they provide evidence of a program’s generalisability and applicability to all CALD backgrounds nor specific sub-groups—which ultimately limits potential usability and potential positive impacts of these programs and findings. It is also noted that the acronym or label CALD is inconsistently defined in literature [30] and can be problematic due to perpetuating and normalizing the separation from the dominant cultural group and its failure to sufficiently acknowledge the intersectoral challenges faced by people and communities ‘collected’ under this term [2, 31]. There is an abundance of literature describing frameworks for developing mainstream program evaluation and literature describing sexual health program development, however, there is scant academic literature on program development with young people from CALD backgrounds and even less reporting evaluation processes and outcomes. For example, one of the few studies identified describing the development of sexual health programs in CALD communities in Australia, described how a Muslim school introduced a sexual health program into their curriculum; however, it did not report on program evaluation [32]. Such information is needed to ensure content is current, culturally safe and acceptable, age and developmentally appropriate, and responsive to young people’s needs to enhance uptake and effectiveness. Information on evidence-based sexual health education program evaluation is needed to assist in the proposed development of novel and engaging sexual health programs for youth [33]. There is a fiscal and ethical imperative to evaluate health promotion resources, especially regarding sensitive topics and focussing on vulnerable priority groups.

## This study

This scoping review identifies studies from organizations in Australia, Canada, NZ, and the United Kingdom (UK) (higher-income countries with similar health systems, migration patterns and socioeconomic status) that evaluated SRH programs for young people (aged 10–24, per WHO [34]) from CALD backgrounds. Through a systematic search of academic and grey literature, this review maps study characteristics to guide health promotion policy. It also informs the development of evidence-based SRH evaluation tools, identifies knowledge gaps, and suggests future research to improve SRH outcomes for young people.

## Objectives

The aim of this review was to identify the content developed for sexual health programs targeting young people from CALD backgrounds living in Australia, Canada, NZ and the UK and any program evaluation strategies applied. Specifically, the review aimed to address the following questions regarding program evaluation:

- To what extent are the content and resources in Australia, Canada, NZ and the UK evaluated?
- Who conducts and participates in the sexual health program evaluation?
- What are the guiding theoretical frameworks within the program?
- What are the guiding questions within the program development regarding SRH?
- What is the contribution of young people, their families, and communities/peers at any point in the evaluation cycle and program development?
- What cultural considerations have been considered and/or used to inform evaluation design?

## Methods

The review followed PRISMA-ScR guidelines for scoping reviews [35]. The framework was designed by the first author in consultation with co-authors from diverse disciplines (nursing, psychology, public health and CALD health promotion researchers) and cultural backgrounds. All authors have experience working with diverse populations, particularly at the cultural interface, focusing on refugee and migrant health, health equity, and culturally safe sexual health education. Data extraction was structured to answer the research questions, focusing on program content, evaluation strategies, theoretical frameworks, and cultural considerations. Extracted data included: authors, publication year, country, study aims, setting, evaluation framework, participants, outcomes, and key findings. This ensured systematic analysis of the literature. Study quality and risk of bias were not assessed, as the review focused on evaluation strategies and program content, not methodological quality.

### Eligibility criteria

The review included Australia, Canada, NZ and the UK for several reasons. Firstly, these countries have similar immigration-driven population growth. They also have publicly funded universal health systems with a focus on equity, enabling access to sexual health services for diverse populations. Additionally, all four countries are aligned in their provision of evidence-based, comprehensive sexual health services and education. Finally, they are all English-speaking nations, which supports consistency in service delivery and literature accessibility.

**Participants:** participants were considered peer-reviewed or grey literature (There was no limitation to the date range) describing evaluation of content for sexual health programs and resources targeting young people from CALD backgrounds living in Australia, Canada, NZ and the UK.

**Concept:** the concept explored what strategies and frameworks were used to assess the effectiveness and relevance of the sexual health program content and resources for young people of CALD background (defined as people who were born overseas or direct descendants of immigrants). Programs included provided and disseminated information about sexual health to young people of CALD background. The content refers to the topics covered in the programs. Resources included the staff and trained personnel (including peer-mentors and leaders), teaching aids, and take-home fact sheets *etc.* The evaluation includes evaluation frameworks, protocols, feedback forms and questionnaires, and government and non-government stakeholder policies, information, and reports.

**Context:** context is the place where the sexual health program was conducted. It includes young adult drop-in centers, sexual health clinics, medical clinics, settlement and migration services, school and community-based sexual health education programs, and online information.

### Exclusion criteria

Exclusion criteria included academic papers or grey literature whose outcome variable for program effectiveness was epidemiological and described the sexual health of young people in terms of the number of unplanned pregnancies, the number of STI/HIV/BBV diagnosed, the number of people visiting clinics, and available services. These are indirect measures of content effectiveness but not necessarily the acceptability of the content and resources. Literature that described the process of establishing sexual health programs but omitted discussing evaluation were also excluded. Additionally, literature was excluded if it only described the development phase of the program without evaluating its content and/or resources postimplementation. Literature describing sexual health programs for First Nations Peoples was also excluded due to unique cultural features within the project target populations. Finally, countries other than the four included were excluded due to differing migration histories, healthcare systems (such as the United States, which has a non-universal, privatized system that presents unique barriers), and socioeconomic backgrounds (such as either being non-English-speaking or low- and middle-income countries. These exclusions were made to maintain consistency in language, health infrastructure, and the availability of comparable literature.

### Search strategy

Databases searched were APA PsychArticles, APA PsychInfo, CINAHL, Education Research Complete, ERIC, Health Source: Nursing/Academic Edition, Psychology and Behavioral Science Collection, and Sociology Source Ultimate, Medline, ScienceDirect, Scopus, Web of Science, and Google. The search string was determined by inserting sexual health, SHL, assessment, and young people into the search bars of Google, EBSCOhost, and MeSH. The search string (‘sexual health’ AND (resources OR support OR services)) AND (assessment OR evaluation OR report) AND (young OR youth OR adult) was adapted as necessary for each of the databases. The search was limited to the titles and abstracts and occurred 24 September 2021.

An ‘Advanced Search’ on Google (not Google Scholar) was conducted for government and non-government agency domains (including generic domai-ns such as .org and .com that are widely used by non-profit organizations and private entities and are not country specific but checked by the research team). A government and non-government organization domain search, first for program evaluation and next for reports, were conducted for each country for a total of 16 searches. The search for each domain was: ‘program evaluation’, the exact phrase ‘sexual health’, and any of the words ‘young youth adult’. No other filters were applied the advanced search. The advanced searches on Google were conducted 26 September 2021.

Author five (an expert in CALD sexual health and wellbeing) provided a list of 13 key peer stakeholder/community-based organizations which provide sexual health services and support to CALD communities in Australia to contact to request their insights as peer workers in this space. The organizations were emailed and asked if they could contribute any information on content and resource evaluation to the review.

### Screening and data charting

The screening procedures were reviewed by authors one and three. Discrepancies were discussed between authors one and three in first instance, and then taken to the wider research team (all authors) for any unresolved (for which there were none).

## Author positionality statement

The authors of this study include people with lived experience of being migrants living in Australia (from various parts of the world, e.g. Africa, Asia, Europe, North America), as well as clinicians and academics. There is experience working in SRH with members of culturally and racially marginalized communities experience ranging from 3 to 20 years (average 17 years), and spanning a wide range of aligned disciplinary (*e.g.* Nursing & Midwifery, Public Health, Clinical Psychology, Health Psychology, Medicine) and research (e.g. health promotion, global health, refugee and migrant health, health equity, health systems, marginalized populations, relationship and sexuality education, cultural safety) areas of expertise. Co-authors identify with the following ethnicities (Chinese, Ethiopian, Native American, American European, Celtic American, Irish, Caucasian, Celtic Australian, and Australian). This research has been conducted in partnership with peak community-based body which supports the sexual health and wellbeing of people from culturally and racially marginalized backgrounds in Australia.

## Results

**Figure 1:**
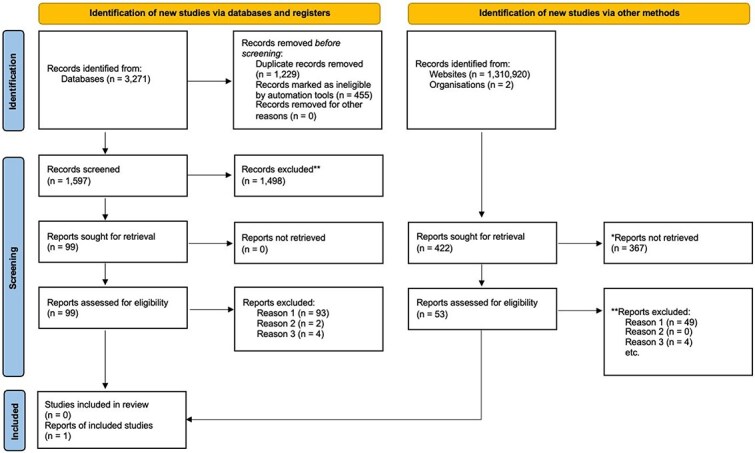
Prisma ScR flow chart. *Note.* Reports not retrieved include 65 duplicates identified when the advanced Google hits were merged. Reason 1 = did not fulfil all the inclusion criteria, Reason 2 = unable to retrieve full text; Reason 3 = Report for indigenous peoples.

A total of 3271 papers were identified through the academic databases, which were imported to Endnote X9.3.3 referencing software (https://endnote.com), visual inspection (authors screening based on title) removed 1684 duplicates, leaving 1397 papers for screening. During title screening, 1298 were removed leaving 99 papers. These were then screened for evidence that the content and resources were assessed and if it was unclear, the full-text was read. No papers satisfied the inclusion criteria (see Fig. 1).

The advanced Google search identified 1 310 920 items. The first 30 hits for each advanced Google search were screened as these were generally the most relevant and not duplicates. Thus, a total of 420 sites were screened. When the Google searches were pooled, 65 duplicate results were identified and removed, leaving 355 reports. The documents were downloaded and examined (reviewed by title, abstract/summary, and full text as necessary); however, none satisfied the inclusion criteria for review (see Fig. 1).

A search was conducted on the 13 organizations provided by author five, which identified a further seven organizations. The 20 organizations were emailed and invited to contribute to the research. Two organizations provided information on program evaluation: one was an external assessment of sexual health factsheets which were translated in 17 different languages but was not developed to target young people and therefore was not included in the review. The other organization provided information *via* email which described the evaluation of content for a sexual health program targeting young people and was included in the review (see Fig. 1).

No academic literature nor grey literature reports were identified that described the assessment of sexual health program content or resources targeting young people of CALD backgrounds in Australia, Canada, NZ, or the UK. One article of grey literature, an email from a community service (personal communication with Piergiorgio Moro, Coordinator at CEH) [36] providing cultural competence training to healthcare professionals and multicultural health resources, described the process of an informal evaluation for the resources developed for this group [36]. They reported that it is standard practice to ask training participants to complete an evaluation (however no details were provided). Resources were evaluated by community members during their development (see Table 1—including demographic details and characteristics of the resources) and resource users can provide feedback; however, feedback is not actively sought. Young people were not specifically mentioned as active contributors to the evaluation of the sexual health resources. Resource usefulness and relevance is primarily determined by how often a resource is used [36]. Furthermore, a full evaluation of a program offered by this organization is only completed when requested by a stakeholder. Limited findings support suspected paucity of research in this space and historical underreporting or lack of formally evaluated health promotion consistent with a lack of identified eligible studies in this review.

**Table 1:** List of demographics and resource characteristics community members may comment on used during resource development. [36]

Demographics
Cultural background
Age
Job
Education
Characteristics of the resource^*^
Look of the resource
Cultural relevance
Readability of the text
Language easy to understand
Translation: Does it make sense?
Ease of comprehension
Errors in the text?
Clarity of terms
Suggest Alternative terms
Other comments

^*^Respondents are asked to explain their response.

## Discussion

All young people have a right to age-, developmentally-, and culturally appropriate sexual health information. However, little is known about the evaluation processes that ensure content and resources are appropriate and updated for young people from CALD backgrounds. This scoping review aimed to map the evaluation frameworks and processes used to assess sexual health programs and resources targeting this group across four high-income countries with similar healthcare systems and immigration patterns. However, we found no evidence of these evaluation frameworks and processes published in publicly available academic literature, government reports, or non-government documents. One organization provided unpublished information on request that demonstrates CALD communities are involved in the planning and development of their sexual health programs, a strategy known to improve cultural relevance [37]. However, systematic evaluation of the program was limited and *ad hoc*.

This review reveals a significant gap in evidence, possibly due to a desire for confidentiality in evaluation processes, challenges in publishing frameworks, or limited research funding for evaluating sexual health programs for CALD communities. Service providers may prioritize the design and delivery of evidence-based programs over evaluation, focusing on keeping content relevant to emerging evidence and societal changes. However, our lack of findings suggests limited resources, systems, or understanding of the importance of evaluation. Ultimately, more evaluation and dissemination of results are needed.

Evaluation frameworks and processes are widely published in facilitation, organizational psychology, and educational journals. The diverse contexts which give rise to SRH literacy for CALD peoples presents a challenge in determining a gold-standard evaluation process for assessing CALD related programs and resources. This diversity also means that it is unlikely to be a single assessment tool for long term evaluation and monitoring of the topic content and support material. However, common features of evaluation frameworks include the focus on specific aspects of program and that theoretical frameworks guide the evaluation [38–40]. The features of an effective health program evaluation are that they monitor the program’s effectiveness and efficiency [40] through long-term repeated monitoring [39] from participants’ point of view and their experiences [41]. Participant-focused evaluation ensures that their voices are central to any modification [42] and is in-line with a person-centered approach to care [43] and intervention [44]. Thus, gold-standard of CALD SRH evaluation is i.e. an accurate representation of the experiences of the participants and is process is guided by theoretical frameworks.

A framework which fills the gold-standard criteria is the Viability Model [45]. The Viability Model describes a process of discovering the extent to which the program was helpful or harmful, thereby revealing what was relevant and culturally appropriate to the participant. Such information could pinpoint the content and learning materials which need to be modified, removed, or included. An alternative is framework is the non-adoption, abandonment, scale-up, spread, and sustainability (NASSS) framework [46]. Although the NASSS explores the impact of technology, it may be modified to evaluate and modify program materials, so the messages remain relevant and accessible for participants [47].

## Implications for research and practice

There is an urgent need to develop frameworks and guidelines for evaluating sexual health programs for young people from CALD backgrounds. It is essential to address the paucity of evidence on the effectiveness and efficiency of these programs and to ensure the development of clear, evidence-informed evaluation tools for service providers to embed within programs in the future.

## Future research

Our findings indicate a salient gap in knowledge on how or even if organizations evaluate the content and resources used in sexual health programs for young people from CALD backgrounds. This calls for research to inform development of an age, developmentally, and culturally appropriate theoretical framework for evaluation along with research to explore how best to implement this framework into sustainable standard practice and ongoing quality improvement activities.

It is recommended that evaluation outcomes extend beyond epidemiological descriptions of behaviour change to understanding (i) patterns of engagement in the content and resources, (ii) how engagement in the resources affects sexual behaviours and mental health, (iii) factors which mediate and moderate the relationship between resource engagement and sexual behaviour such as age, adjustment to resettlement, competency in English, trauma experienced during the forced migration journey, and years in Australia. Further, there is little understanding about how young people from CALD background’s engagement in program development and evaluation affects their long-term sexual health choices, family and peer relationships, cultural and religious beliefs, wellbeing, and mental health. A holistic understanding the effect of sexual health information on the social and spiritual life of young people from CALD backgrounds, may help community and health workers to work more sensitively and effectively with young CALD people including overcoming the challenges of stigma and cultural norms preventing these young people achieving SHL [2, 48, 49], including regarding contemporary sexual health contexts (e.g. PrEP) [50–52]. Further, integration of bilingual health workers may help to support and enhance these processes [53].

Greater understanding of why organizations do not publish or have evaluation reports publicly available is also needed. Strategies to address the barriers faced by organizations and service providers to implement evaluation frameworks and processes as part of standard practice are needed.

## Limitations

Despite these suggestions, there is an urgent need to develop valid and reliable frameworks and guidelines for evaluating sexual health for young people from CALD background. It is essential to address the paucity of evidence on the long-term evaluation of these programs and to ensure the development of clear, evidence-informed evaluation tools for service providers. Additionally, a growing body of work highlights the limitations of the CALD term, with recent research acknowledging the need for more precise and reflexive terminology to better capture the complexity of migrant experiences in research and public health practice. While the CALD label was used here for brevity and to allow word space for in-depth discussion of findings, its definition remains inconsistent in the literature [30], and concerns have been raised about its divisiveness and lack of recognition of intersectoral challenges [31, 54–56].

## Conclusion

Young people from CALD backgrounds have important and timely need for sexual health information, however information needs to be presented in culturally safe and age/developmentally appropriate ways. This study identified the concerning lack of evaluated programs and the implication of an ‘urgent call to action’ to address this. This scoping review, conducted to determine how or if organizations evaluate the cultural and age appropriateness of sexual health content targeting young people from CALD backgrounds in Australia, Canada, NZ and the UK, found that literature reporting on evaluation frameworks, processes and outcomes specific to this population was minimal. There appears to be some systematic review of content and resources provided during formal education sessions. However, if young people from CALD backgrounds are to have access to evidence-informed age-, developmentally-, and culturally appropriate sexual health information, organizations developing and providing programs need to work with researchers and policymakers to develop theoretical frameworks for evaluation, along with implementation frameworks to ensure these programs are contemporary and meet the needs of this heterogeneous group of young people.
